# Genotype diversity of *Trypanosoma cruzi* in small rodents and *Triatoma sanguisuga* from a rural area in New Orleans, Louisiana

**DOI:** 10.1186/s13071-015-0730-8

**Published:** 2015-02-24

**Authors:** Claudia P Herrera, Meredith H Licon, Catherine S Nation, Samuel B Jameson, Dawn M Wesson

**Affiliations:** Department of Tropical Medicine, Vector-Borne Infectious Disease Research Center, School of Public Health and Tropical Medicine, Tulane University, 1440 Canal Street, Rm. 1824, New Orleans, LA 70112 USA

**Keywords:** *Trypanosoma cruzi*, *Triatoma sanguisuga*, Genotyping, *T. cruzi* DTUs, Rodents, United States

## Abstract

**Background:**

Chagas disease is an anthropozoonosis caused by the protozoan parasite *Trypanosoma cruzi* that represents a major public health problem in Latin America. Although the United States is defined as non-endemic for Chagas disease due to the rarity of human cases, the presence of *T. cruzi* has now been amply demonstrated as enzootic in different regions of the south of the country from Georgia to California. In southeastern Louisiana, a high *T. cruzi* infection rate has been demonstrated in *Triatoma sanguisuga*, the local vector in this area. However, little is known about the role of small mammals in the wild and peridomestic transmission cycles.

**Methods:**

This study focused on the molecular identification and genotyping of *T. cruzi* in both small rodents and *T. sanguisuga* from a rural area of New Orleans, Louisiana. DNA extractions were prepared from rodent heart, liver, spleen and skeletal muscle tissues and from cultures established from vector feces. *T. cruzi* infection was determined by standard PCR using primers specific for the minicircle variable region of the kinetoplastid DNA (kDNA) and the highly repetitive genomic satellite DNA (satDNA). Genotyping of discrete typing units (DTUs) was performed by amplification of mini-exon and 18S and 24Sα rRNA genes and subsequent sequence analysis.

**Results:**

The DTUs TcI, TcIV and, for the first time, TcII, were identified in tissues of mice and rats naturally infected with *T. cruzi* captured in an area of New Orleans, close to the house where the first human case of Chagas disease was reported in Louisiana. The *T. cruzi* infection rate in 59 captured rodents was 76%. The frequencies of the detected DTUs in such mammals were TcI 82%, TcII 22% and TcIV 9%; 13% of all infections contained more than one DTU.

**Conclusions:**

Our results indicate a probable presence of a considerably greater diversity in *T. cruzi* DTUs circulating in the southeastern United States than previously reported. Understanding *T. cruzi* transmission dynamics in sylvatic and peridomestic cycles in mammals and insect vectors will be crucial to estimating the risk of local, vector-borne transmission of *T. cruzi* to humans in the United States.

## Background

Chagas disease is an anthropozoonosis caused by the protozoan parasite *Trypanosoma cruzi* and represents a major public health problem in Latin America with a burden of disease five times higher than malaria as measured by DALYs [1]. Due to human migration from Chagas endemic countries, this disease, which has long been considered a disease of the poor in Latin America, has crossed borders and become a public health problem in non-endemic regions [2]. Although the United States (US) was initially defined as non-endemic for Chagas disease due to the rarity of human cases [3], *T. cruzi* has now been amply demonstrated as enzootic in different regions of the south of the country from Georgia to California [1,4].

More than 130 triatomine insect species have been described throughout the Americas. Eleven of these species have been found naturally infected with the parasite in the US and are considered potential Chagas disease vectors. Of these species, *Triatoma sanguisuga* and *Triatoma gerstaeckeri* are considered the most important vectors in the southeastern and southcentral US, respectively [5-7]. Many animals can be infected by and serve as reservoirs for *T. cruzi*. At least twenty-four species are recognized as natural sylvatic hosts for *T. cruzi* in the US including woodrats, opossums, raccoons, armadillos, and skunks [1,8]. *T. cruzi* transmission in the US was previously thought to be associated only with sylvatic or rural environments. However, Beard et al. [9] reported a domestic transmission cycle in southern Texas in which six dogs tested positive for *T. cruzi* infection and three died of Chagas disease. These findings suggest that the typically sylvatic *T. gerstaeckeri* may have passed the parasite from a sylvatic, enzootic cycle to a peridomestic cycle in dogs, which in this case may play an important role in supporting peridomestic transmission of *T. cruzi* [9] and increased risk of exposure for humans. In contrast to the findings in southern Texas, a recent study of factors associated with peridomestic *T. sanguisuga* demonstrated the lack of association between dog ownership and the presence of *T. sanguisuga* in and around houses [10]. Although the full epidemiologic role of dogs has not been well established in the US, they could play an important role in peridomestic and domestic transmission of *T. cruzi* as has been reported in Latin American countries [11].

Since the first autochthonous human case of Chagas disease was described in 1955, only 22 human cases have been reported that presumably were acquired via local vector-borne transmission in the US [1,12,13]. One of these cases was a woman from rural New Orleans, Louisiana, who was infected through the only local vector, *T. sanguisuga.* More than half (56%) of twenty dead adult triatomines collected inside and around the woman’s house were positive for *T. cruzi* by PCR analysis [14]. A more extensive study of 298 *T. sanguisuga* specimens showed 180 (60.4%) were positive for *T. cruzi* by PCR [15]. This prevalence was similar to that reported in *T. gerstaeckeri* collected in Texas during the years 2005–2006 [16].

Due to its high genetic variability, *T. cruzi* has been classified into six discrete typing units (DTUs) named TcI-TcVI, and a possible seventh one named Tcbat. Each DTU has been associated with different transmission cycles, hosts, and geographical distributions throughout the Americas [17]. Recent reports concerning the genetic characterization of *T. cruzi* stocks from the US have only demonstrated the presence of TcI (primarily in opossums and *T. sanguisuga*) and TcIV (primarily in raccoons, domestic dogs, and other mammals and in *Triatoma* species). Both DTUs have been described in domestic and wild transmission cycles [4,18]. Recent studies in Mexico report the presence of five of the six DTUs from a sample of more than 300 triatomine specimens including *T. dimidiata, Meccus pallidipennis, M. longipennis* and *T. barberi.* The samples were collected in domestic and peridomestic areas in a *T. cruzi* hyperendemic area in Veracruz and in another area in Michoacan de Ocampo [19,20]. These observations in areas where only TcI had been reported previously suggest that there may be a much greater diversity of *T. cruzi* DTUs not only in Mexico, but also in other parts of North America. Given these findings, we conducted a study to identify the diversity of circulating *T. cruzi* DTUs in captured rodents and *T. sanguisuga* from in and around the home of the autochthonous Chagas disease case previously reported in rural New Orleans, Louisiana.

## Methods

### Rodent and vector collection area

The collection area for both rodent and *T. sanguisuga* samples was the interior and immediate surrounding ecotopes of the residence of the first autochthonous human case of Chagas disease reported from New Orleans in 2006 [14] located on the West Bank of Orleans Parish, Louisiana [10]. The residence was situated within a zone where previously, a high prevalence of *T. cruzi* (60.4%) was observed in *T. sanguisuga* [15]. Tomahawk and H. B. Sherman live-animal traps were placed for targeted trapping of rodents based on homeowner complaint and evidence of rodent activity. The domestic and peridomestic areas were allowed to be naturally recolonized by rodents until the homeowner requested that trapping resume. Trapping was conducted in four rounds beginning in September 2009, June 2011, May 2012, and February 2013. To obtain information on the organ distribution of the different *T. cruzi* strains to be identified, samples of liver, spleen, heart, and skeletal muscle were collected at necropsy and stored at −20°C until testing. *T. sanguisuga* were collected from woodpiles surrounding the home. Teams of trained individuals methodically deconstructed each woodpile while searching for adult and immature stages of *T. sanguisuga*.

### Isolation and identification of *T. cruzi* from vectors

The intestinal contents from *T. sanguisuga* collected during 2012–2013 were analyzed by direct microscopic observation to identify flagellate organisms resembling *T. cruzi*. Feces from vectors with positive observation for flagellates were cultured in NNN (Novy, McNeal and Nicolle) medium supplemented with RPMI1640 (Roswell Park Memorial Institute), 20% fetal bovine serum and 80 mg/ul gentamicin at 28°C. Cultures were checked every third day until *T. cruzi* parasites were detected.

### Diagnosis of *T. cruzi* infection

DNA was extracted from samples, including heart, liver, spleen and skeletal muscle obtained from each rodent, feces from insects, and isolated parasites from *T. sanguisuga* feces maintained in culture using DNeasy Blood & Tissue Kit (QIAGEN, Valencia, CA). All DNA samples were tested by PCR for the presence of *T. cruzi*. DNA from four different reference strains of *T. cruzi* maintained in our laboratory was also extracted for use as positive controls. The reference strains were chosen to correspond to four different DTUs: Sylvio X10 (DTU TcI), Esmeraldo (DTU TcII), CAN III (DTU TcIV) and SC43 cl1 (DTU TcV). In addition, DNA was extracted from tissues of two un-infected mice from Tulane University’s Department of Comparative Medicine. A no DNA template negative control was also used in the PCR.

Diagnostic PCR was performed on all samples using two different molecular markers for *T. cruzi*: the minicircle variable region of the kinetoplastid DNA (kDNA) using the primers S35/S36 and the highly repetitive genomic satellite DNA (satDNA) using the primers TcZ1/TcZ2 (Table 1).

**Table 1:** **Primers used for the detection and genotyping of** ***Trypanosoma cruzi.*** **Primer**

	**Sequence**	**Target**	**Reference**
S35	5-’AAA TAA TGT ACG GGK GAG ATG CAT GA	Minicircle variable region of kDNA	[21]
S36	5’-GGG TTC GAT TGG GGT TGG TGT		
TcZ1	5’-CGA GCT CTT GCC CAC ACG GGT GCT	Nuclear satellite DNA	[22]
TcZ2	5’CCT CCA AGC AGC GGA TAG TTC AGG		
Tc1	5’-ACA CTT TCT GTG GCG CTG ATC G	Miniexon intergenic region	[23]
Tc2	5’-TTG CTC GCA CAC TCG GCT GCA T		
Tc3	5’-CCG CGW ACA ACC CCT MAT AAA AAT G		
Tr	5’-CCT ATT GTG ATC CCC ATC TTC G		
Me	5’-TAC CAA TAT AGT ACA GAA ACT G		
D71	5’-AAG GTG CGT CGA CAG TGT GG	D7 divergent domain of the 24Sα rRNA gene	[24]
D72	5’-TTT TCA GAA TGG CCG AAC AGT		
V1	5’-CAA GCG GCT GGG TGG TTA TTC CA	Size-variable domain of the 18S rRNA gene	[25]
V2	5’-TTG AGG GAA GGC ATG ACA CAT GT		

Amplification was performed in a final volume of 25 μL. To increase the Taq polymerase performance a mixture of 18 μL of Apex™ Hot start 1.0X PCR Master Mix (Genesee Scientific, USA) was used with 1.5 μM of each primer set (S35/S36 or TcZ1/TcZ2) and 20 ng of template DNA. For kDNA amplification, products were generated in a C1000 Touch™ Thermal Cycler (Bio-Rad, USA), and the cycling conditions were as follows: an initial denaturation step at 95°C for 5 min, followed by 30 amplification cycles of 95°C for 1 minute, 60°C for 1 min, and 72°C for 1 min with a final extension step at 72°C for 5 min [26]. Cycling conditions for satDNA were as follows: an initial denaturation step at 94°C for 5 minutes was followed by 40 cycles of 94°C for 45 seconds, 68°C for 1 minute, 72°C for 1 minute and a final extension step at 72°C for ten minutes [27]. PCR products were separated on 2% agarose gels and visualized with ethidium bromide. Those samples demonstrating banding at 330 bp for kDNA and/or 188 bp for satDNA were considered *T. cruzi*-positive and selected for further genotyping.

### Molecular identification of *T. cruzi* DTUs

*T. cruzi* genotyping was accomplished using three different molecular markers. The first marker tested was the intergenic region of the miniexon gene of *T. cruzi*. This was analyzed by multiplex PCR using five primers: Tc1 (DTU TcI), Tc2 (DTUs TcII-V-VI), Tc3 (DTUs TcIII-IV), Tr (*T. rangeli*), and Me (common oligonucleotide downstream from the most conserved part of the miniexon gene) (Table 1). PCR was performed in a 25 μL reaction mixture, containing Apex™ Hot start 1.0X PCR Master Mix (Genesee Scientific, USA), 1.5 μM of each primer and 40 ng of DNA extracted from tissue samples or 20 ng of DNA extracted from triatomine feces or parasites from culture media. Amplification products were generated in a C1000 Touch™ Thermal Cycler (Bio-Rad, USA) using 35 cycles of 5 min at 94°C, 30 seconds at 94°C, 30 seconds at 55°C, and 30 seconds at 72°C followed by a final extension step at 72°C for 7 minutes. This technique distinguishes TcI from other DTUs and groups the remaining DTUs into two groups based on the size of the amplified product. In this reaction, TcI has an expected fragment of 200 bp; TcII, TcV, and TcVI have an expected fragment of 250 bp; and TcIII and TcIV have an expected fragment of 150 bp. Samples determined to be TcII, TcV, or TcVI were further classified by PCR amplification of the D7 divergent domain of the 24Sα rRNA gene and the size-variable domain of the 18S rRNA gene. The primers used were D71/D72 and V1/V2, respectively (Table 1).

### Sequencing and editing of the miniexon gene intergenic region

DTU identification was confirmed by sequencing PCR amplicons from the miniexon intergenic region after purification using a PureLinkTM Quick PCR purification kit (Invitrogen®). For samples that showed more than one amplification band for a molecular marker, each band was excised and recovered using ZymocleanTM Gel DNA Recovery Kit (Genesee Scientific®) according to the manufacturer’s instructions. All samples were sequenced by GENEWIZ, Inc (South Plainfield, NJ) using Applied Biosystems’ BigDye version 3.1 and Applied Biosystems’ 3730xl DNA Analyzer. The sequences were edited manually using MEGA version 5 software [28]. A total of 15 sequences of sufficient quality were obtained for analysis (S26MS NOLA, S18MH NOLA, S26ML NOLA, S38MH2 NOLA, S14MH NOLA, S52RH2 NOLA, S38MH1 NOLA, S52RH1 NOLA, S6MM NOLA, S6ML NOLA, S7MH NOLA, S9MS NOLA, S29MH NOLA, S29ML NOLA, and S26MHNOLA (Accession numbers: KM376435- KM376449)).

### Phylogenetic analysis

Maximum-likelihood (ML) method was performed for the phylogenetic analysis [28,29]. A total of 27 sequences were used, including 15 sequences corresponding to *T. cruzi* from the collected rodent tissue samples and 12 reference sequences of the DTUs TcI, TcII, TcIV, TcV, TcVI. The reference sequences were obtained from the GenBank database: USAOPOSSUMcl2 (Accession number: JQ581510.1), 92090802Pcl1 (JQ581481.1), USAARMAcl3 (JQ581509.1), Gal61C (EU626731.1), Tu18 (AY367125.1), AF1Cl7 (FJ463161.1), CANIII (AY367123.1), MN (AY367128.1), VSC (FJ463159.1), SN6C (AM259471.1), Tc167(AM259480.1). The alignment was adjusted to obtain the best-fitting model based on the Akaike Information Criterion (AIC) [28]. The best fit substitution model selected for this data set was determined to be GTR (general time-reversible model) + I (invariable sites proportion) + G (a gamma disturbed rate of variation among sites). The trees and the robustness of the nodes were evaluated by bootstrap on 1000 replications by heuristic search. Trees were obtained automatically by applying Neighbor-Join and BioNJ algorithms and then selecting the topology with superior log likelihood value using MEGA version 5 software [28].

### Ethical approval

All animal procedures were approved by the Tulane Institutional Animal Care and Use Committee (Protocol No. 4130).

## Results

### Identification and isolation of *T. cruzi* from vectors

Between 2012 and 2013, twelve specimens of triatomines were collected from peridomestic woodpiles. Species was determined using morphologic characteristics [30]. All were identified as *T. sanguisuga* and both adult and nymph insect stages were kept alive until feces could be collected for microscopic examination.

**Figure 1:**
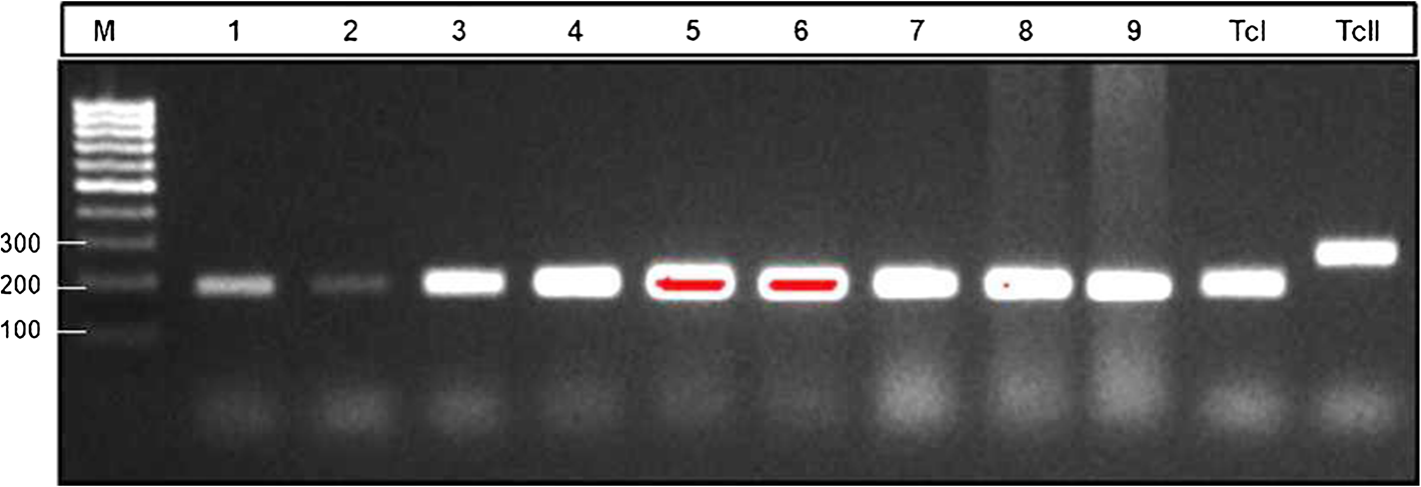
**Molecular characterization of the flagellate forms from** ***T. sanguisuga*** **feces and** ***T. cruzi*** **strain isolates by amplification of the mini-exon intergenic region.** (1–3) DNA from flagellate forms in feces: WB1F, WB2F, WB3F (4–6) DNA from *T. cruzi* strain isolates from feces of samples 1–3 respectively, (7–9) DNA from *T. cruzi* strain isolates WB4, WB758, WB759. DNA from references strains: TcI (Sylvio 10X), TcII (Esmeraldo). Agarose 2.5% gel.

Of the twelve collected specimens, eight were positive for flagellated parasites by direct microscopic observation and six strains were successfully isolated in culture media. All six strains were confirmed as *T. cruzi* by diagnostic PCR. The genotyping analysis showed that all six strains belong to the TcI DTU (Figure 1).

### Genotyping of *T. cruzi* from rodent tissue samples

A total of fifty-nine wild and peridomestic rodents were captured, specifically 44 mice and 15 rats. Using morphological characteristics [31], three species were identified: *Peromyscus gossypinus* 40/59 (67.8%), *Mus musculus* 4/59 ( 6.8%), *Neotoma floridana* 15/59 (25.4%).

Using two different molecular markers, the minicircle variable region of the kinetoplastid DNA (kDNA) and the highly repetitive genomic satellite DNA (satDNA), a total of 34/44 (77.2%) mice (32 *P. gossypinus* and two *M. musculus)* and 11/15 (73.3%) rats (*N. floridana)* were positive for *T. cruzi*, corresponding to an overall rodent infection rate of 76% (Table 2). The genotype of *T. cruzi* was determined from tissue samples for positive rodents using the miniexon gene. From a total of 45 positive rodents, differentiation among DTUs was achieved in 23 rodents: 20 mice (44.4%) and 3 rats (6.6%). A total of 18 of these rodents (78.2%) were found to be infected only with TcI. Single infections with non-TcI genotypes were detected in 2/23 (9%) rodents. Mixed infections with different genotypes were detected in 3/23 (13%) rodents. These samples were further analyzed by ribosomal markers and sequencing to specify the infecting non-TcI DTU. Surprisingly, within these samples TcII was identified in two mice displaying single infection (9%). It was also detected in 2/23 (9%) of mice in association with TcI and 1/23 (4%) of those with TcIV. Considering the data together, a total of 19/23 (82%) rodents were infected with TcI, 5/23 (22%) with TcII, and 2/23 (9%) with TcIV (Table 3).

**Table 2:** ***Trypanosoma cruzi*** **infection in rodents identified by species**

**Host**	**Species**	**Infection rate**
Mouse (n = 44)	*Peromyscus gossypinus*	32 (73%)
	*Mus musculus*	2 (5%)
Rat (n = 15)	*Neotoma floridana*	11 (73%)
**Total (N = 59)**		**45/59 (76%)**

**Table 3:** **Frequency of** ***T. cruzi*** **DTUs and mixed infections in infected rodent samples successfully genotyped**

**Host**	**DTU and Mixed infection frequency**			
	**TcI only**	**TcII only**	**TcI-TcII**	**TcII-TcIV**
Mouse (n = 20)	16 (80%)	2 (10%)	1 (5%)	1 (5%)
Rat (n = 3)	2 (67%)	ND	ND	1 (33%)
**Total (N = 23)**	**18 (78%)**	**2 (9%)**	**1 (4%)**	**2 (9%)**

ND: Not detected.

### Mini-exon intergenic region sequencing and phylogenetic analysis

DTU identification was confirmed by sequencing of the mini-exon intergenic region and phylogenetic analysis on a total of 15 sequences. The analysis involved 27 nucleotide sequences with a length of 222 bp. All sequence positions containing gaps and missing data were eliminated. There were a total of 199 positions in the final dataset used to build the phylogenetic tree. The sequences were deposited in the GenBank database of the National Center for Biotechnology Information (NCBI) (http://www.ncbi.nlm.nih.gov/genbank/) (Accession numbers: KM376435- KM376449).

**Figure 2:**
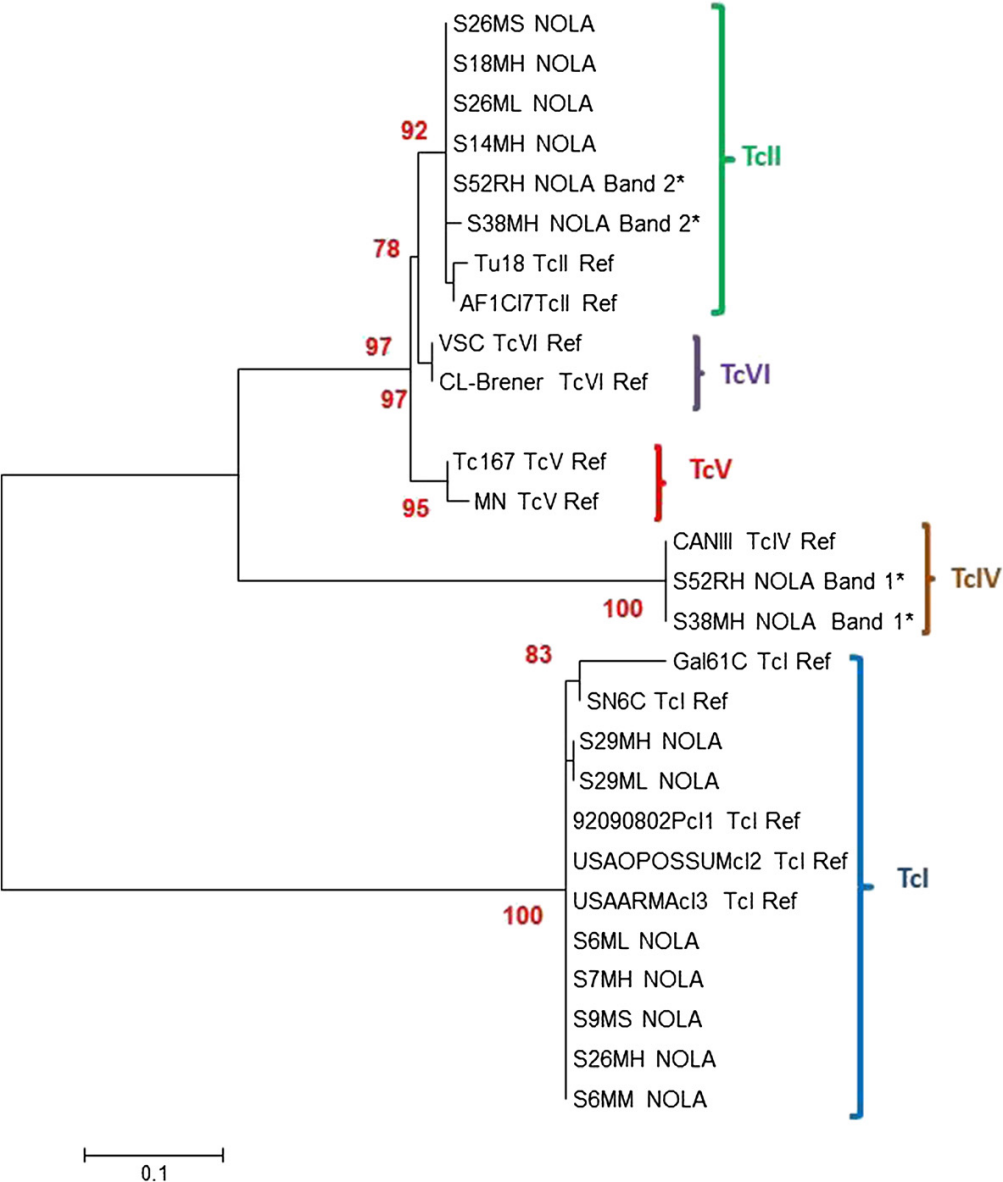
**Molecular phylogenetic analysis by Maximum Likelihood method.** The phylogram depicting the phylogenetic relationships among the 27 *T. cruzi* DNA sequences corresponding to eight rodents, based on Miniexon intergenic gene sequencing. The un-rooted tree with the highest -lnL = 890.6637 is shown, allowing identification of the three different clusters corresponding to three different DTUs in the rodent tissue samples analyzed. Bootstrap values appear on each clustering branch. The last letter in the sample name refers to H: heart, M: skeletal muscle, L: liver, S: spleen. *indicates mice with mixed infections.

We analyzed the phylogenetic relationships among 27 *T. cruzi* DNA sequences corresponding to 15 New Orleans (NOLA) sequences from the collected rodents and 12 reference sequences from known DTUs. Significant bootstrap values were obtained with ML analysis (more than 60%). The unrooted tree with the highest log likelihood value (−lnL = 890.6637) allowed the identification of three main clusters for the rodent tissue samples. The first cluster of six sequences was closely related with the DTU TcII. These sequences showed high genetic identity with the reference sequences, Tul18 and AF1Cl7, with a significant bootstrap of 92%. A second cluster of two sequences corresponded to TcIV and had high genetic identity with the reference sequence CANIII with a bootstrap of 100%. Finally, the cluster of seven samples corresponding to TcI was found to be closely related to TcI sequences from the US with a bootstrap of 100% and slightly less related to Colombian TcI sequences with a bootstrap of 83%. None of the analyzed sequences were grouped with DTUs TcV or TcVI. These data showed a possible extensive diversity of *T. cruzi* DTUs circulating in this endemic area (Figure 2).

### Tissue tropism and DTUs

Table 4 shows the number of the *T. cruzi* DTUs observed in the successfully genotyped rodent tissue samples. Both mice and rats had a higher proportion of parasites in the heart (91%), followed by the liver (26%), skeletal muscle (13%) and spleen (13%). Additionally, each tissue obtained from each rodent was analyzed in order to find a possible association or tropism of the DTU with a specific tissue. Although the majority of the DTUs were found predominantly in the heart, the sample numbers were not sufficient to demonstrate a significant DTU tissue association. We found single infections of the DTUs TcI and TcII in all tissues genotyped in both mice and rats. The highest frequency of *T. cruzi* infection observed in mice was TcI found primarily in the heart (90%) followed by liver and skeletal muscle. Similarly, in rats the highest frequency of *T. cruzi* infection was TcI in the heart (67%); single TcII infection was not found in rats. Mixed infections with TcII and TcIV were only found in the heart of mice and rats while single infections were observed in the other tissues. Contrary to what was expected, in these samples we did not find mixed infection with the DTUs TcI and TcIV (Table 4).

**Table 4:** **Number of the** ***T. cruzi*** **DTUs observed in the rodent tissue samples successfully genotyped**

**Host**	**DTU**	**Tissue**			
		**Heart**	**Liver**	**Skeletal muscle**	**Spleen**
	I Only (n = 16)	14 (88%)	5 (31%)	2(13%)	2 (13%)
**Mouse** (n = 20)	II Only (n = 2)	2 (100%)	ND	ND	ND
	I-II † (n = 1)	1† (100%)	1† (100%)	1† (100%)	1† (100%)
	II-IV (n = 1)	1 (100%)	ND	ND	ND
	Total*	**18/20 (90%)**	**6/20 (30%)**	**3/20 (15%)**	**3/20 (15%)**
**Rat** (n = 3)	I Only	2 (67%)	ND	ND	ND
	II-IV	1 (33%)	ND	ND	ND
	Total**	**3/3 (100%)**	**0/3 (0%)**	**0/3 (0%)**	**0/3 (0%)**
Total (N=23)***		**21/23 (91%)**	**6/23 (26%)**	**3/23 (13%)**	**3/23** **(13%)**

ND: Not detected.

† DTU TcI was found in the heart and TCII was found in the other tissues in the same mouse.

*Total of mice.

**Total of rats.

***Total of rodents.

## Discussion

Despite being home to one of the few US areas with an autochthonous case of human Chagas disease, there exist few studies exploring the genetic variability of *T. cruzi* parasite populations from both insect vectors and mammalian hosts in southeastern Louisiana. In the current study, the molecular analysis from *T. sanguisuga* feces showed a high prevalence (66%) of *T. cruzi* in this vector. This corroborates the prevalence from the same endemic area previously reported by Cesa et al. [15]. Genotyping of the strains isolated from the vectors confirmed the presence of TcI in these samples. Because only twelve *T. sanguisuga* specimens were analyzed, it will be necessary to expand the number of triatomine samples to better evaluate the presence of the other DTUs.

Although rodents have been reported as common hosts for *T. cruzi,* few of these studies have been done in the US. The analysis of different species of small rodents in southern Texas showed a high serological prevalence of *T. cruzi* in the species tested [3]. In our study we report an even higher PCR prevalence of *T. cruzi* in tissues of small rodents with an infection rate of 76%, compared with the infection found in southern Texas plains wood rats (34%), larger mammals such as raccoons (63%), and Virginia opossums (33%) [3,32]. It is unclear why rodents in our study have such a high prevalence of *T. cruzi* infection. It is possible that rodents are more susceptible to *T. cruzi* infection, or that they maintain much higher parasitemias because of their insectivore behavior. It is also unclear what the importance of this high prevalence is from an epidemiological perspective, especially as it relates to the risk for human infection.

Although the first genotyping reports from the US showed the presence of only TcI and TcIV [1,33], our genotyping and phylogenetic analysis indicate the presence of at least three different DTUs (TcI, TcII and TcIV) present in rodents in a small area in rural New Orleans, Louisiana. These results are consistent with the recent findings in southern Mexico where five different DTU’s (TcI, TcII, TcIII, TcIV and TcV) were found in local triatomine collections [19]. Considering that until a few years ago only TcI had been reported in southern Mexico, it is interesting that both TcI and TcV are now being found at the same frequency (27%) in triatomine vectors in this region. These findings do not necessarily represent a change in DTU distribution, but may just be the result of improvements in *T. cruzi* genotyping methods. In addition, in our study we used direct genotyping from biological samples, since culturing parasites prior to genotyping might induce potential bias due to loss of strains or culture selection. In the New Orleans samples, we found a majority contained TcI (82%), which corroborates previous analyses indicating that TcI is the most frequent DTU in raccoons and opossums in the United States [18,33]. Nonetheless, the relatively high frequency of other DTUs found: TcII (22%) and TcIV (9%) indicates the presence of a greater genetic diversity of *T. cruzi* strains in small rodents. In addition, the high *T. cruzi* infection rate we detected suggests the potential for a biological or ecological implication of these small rodents in the peridomestic enzootic cycle of *T. cruzi*. Given the proximity of the collected rodents to humans, further studies should extend collections and analysis to similar rodent populations to assess their epidemiologic importance in autochthonous transmission of *T. cruzi*.

The phylogenetic analysis of the miniexon gene of the NOLA *T. cruzi* sequences showed a strong cluster association with reference sequences of DTU TcII corresponding to Tul18 and AFcl17, with a significant bootstrap value of 92% obtained with ML. A previous phylogenetic analysis of the 24Sα subunit gene found *T. cruzi* isolates from *Triatoma protracta* in California that were grouped in a clade with some TcII and TcVI reference strains, but the different DTUs were not resolved [34]. Our analysis showed a strong and well defined association with the TcII in small rodents, indicating a higher genetic diversity of *T. cruzi* in the US.

It has been shown that different DTUs of *T. cruzi* can be found circulating in the same individual but there is limited evidence that different DTUs can be found in different tissue types within the same host [35-37]. Although DTUs have not shown a strict tropism, a clear association with different types of tissues has been identified [38-40]. In our study the tissue analysis from naturally infected rodents did not show a specific tissue tropism, but they did display mixed infections with DTUs TcI-TcII (different tissues in the same host), and TcII-TcIV (co-infecting tissues in the same hosts). This suggests that there may be a dominance of one of these two DTUs in a tissue-dependent manner.

Epidemiologic evidence suggests a preferential association of *T. cruzi* DTUs TcI and TcII with marsupials and placental mammals, respectively. While TcI was initially associated with marsupials and sylvatic triatomines in wild environments, several studies have shown its presence in domestic environments and linked it with human infections and oral transmission [29,41-43]. TcII is epidemiologically associated with primates, and it is usually found in human infections in the Southern cone countries of South America [44-47]. The presence of TcI, TcII, and TcIV in small rodents in New Orleans may indicate an important role of these mammals in the eco-epidemiological cycle of *T. cruzi* in this region.

This hypothesis will require validation by the examination of additional samples and parasite isolates from the region to better understand the underlying enzootic cycle of *T. cruzi* and how this cycle interfaces with the human population. Identification of TcII in this area for the first time shows that more than TcI and TcIV are present in small mammals in wild and peridomestic locations. Further analysis is necessary to improve the understanding of the molecular epidemiology of *T. cruzi* DTUs circulating in the region.

## Conclusions

Though the number of samples is limited, our results indicate a greater diversity than previously reported in the *T. cruzi* DTUs circulating among triatomine vectors and rodents captured near the house of an autochthonous human Chagas disease case in rural New Orleans. This work should stimulate similar studies in other areas of North America to better understand *T. cruzi* transmission dynamics in sylvatic and domestic cycles among mammals and insect vectors, since such knowledge is crucial to evaluate the transmission risk of *T. cruzi* to humans and their associated domestic animals in the US.
